# Expression of the immune checkpoint molecules CD226 and TIGIT in preeclampsia patients

**DOI:** 10.1186/s12865-024-00603-5

**Published:** 2024-02-07

**Authors:** Cui Li, Haiyan Liu, Zhongliang Duan

**Affiliations:** 1 Clinical Laboratory, Obstetrics and Gynecology Hospital of Fudan University, 419 Fangxie Road, Shanghai, 200011 China; 2 Obstetrics Department, Obstetrics and Gynecology Hospital of Fudan University, Shanghai, China

**Keywords:** Preeclampsia, TIGIT, CD226, CD155

## Abstract

**Background:**

Imbalanced immune responses are involved in developing preeclampsia (PE). We wish to explore the expression and potential changes of immune checkpoint molecules TIGIT, CD226 and CD155 in PE patients.

**Methods:**

The expression of the immune checkpoint molecules TIGIT, CD226 and CD155 in different lymphocyte subpopulations was determined by flow cytometry in 24 patients with PE and compared to 24 healthy pregnant women of the same gestational age as the controls.​Serum CD155 was detected by ELISA in the patients with PE compared to controls.

**Results:**

The percentages of CD4^+^ and CD8^+^ T lymphocytes in the peripheral blood of PE patients were not significantly different from those of the controls, whereas the regulatory T cells (Tregs) in PE patients were significantly lower than those in controls (6.43 ± 1.77% vs. 7.48 ± 1.71%, *P* = 0.0420). The expression of TIGIT and CD226 showed different percentages on CD4^+^ T cells, CD8^+^ T cells and Treg cells. However, the difference in the percentages of TIGIT, CD226 on these T cells between the two groups was not statistically significant. The level of CD155 in peripheral serum of PE patients was 6.64 ± 1.79 ng/ml, which was not significantly different from that in the control group 5.61 ± 1.77 ng/ml, *P* = 0.0505. The present results demonstrate that TIGIT, CD226 and CD155 are not present at altered immune conditions in the peripheral blood of patients with PE, compared with normal pregnant women.

**Conclusion:**

The immune checkpoint molecules TIGIT, CD226 and CD155 are not abnormally expressed in PE patients.

## Background

Preeclampsia (PE) is a condition exclusive to pregnancy that introduces hypertension and proteinuria after 20 weeks of gestation. It’s frequently associated with placental hypoperfusion and multiorgan involvement, causing fetal growth restriction and severe cases that can progress to maternal multiorgan dysfunction, as well as maternal and neonatal death [1]. PE affects approximately 5–10% of pregnant women worldwide [2]. The pathophysiological mechanisms are still incompletely understood, and the involvement of abnormal inflammatory and immune responses [3] is now more widely recognized [3].

T cell immunoglobulin and immunoreceptor tyrosine-based inhibitory motif domains (TIGIT) is an inhibitory immune checkpoint molecule that mainly expressed on immune cells [4]. TIGIT can play roles in mediating inhibitory signaling pathways, and reducing NK cell cytotoxicity and cytokine production and down-regulating effector function of T cell. It is involved in reducing T cell activation, proliferation and cytokine production, enhancing TH2 (helper T cell, TH) immunity, and plays an important role in the negative regulation of immune responses [5]. In pregnant women, the maternal immune system has complex and unique characteristics. However, little is known about the expression and role of TIGIT in pregnant women.

CD226 is a competitive ligand for TIGIT, which is expressed on the surface of most immune cells, particularly T cells, NK cells and monocytes [6]. CD226 is a pro-inflammatory effector co-stimulatory molecule, that competitively binds to the ligand CD155, activates the TCR, promotes TH1-related signaling. It exerts enhanced NK cell cytotoxicity and induces the production of the pro-inflammatory factor interferon gamma by CD4^+^ T cells [7]. It can also induce CD8^+^ T- and NK-cell-mediated immune responses [8]. The expression of CD226 showed a significant changing between pregnant and non-pregnant women [9].

CD155, also known as the poliovirus receptor, belongs to the Nectin family and is widely expressed at low levels on epithelial, endothelial and hematopoietic cells and at high levels on some tumor cells [10]. For example, in patients with multiple myeloma, CD155 expression is correlated with poor prognosis, suggesting that it may be a potential prognostic marker [11]. Serum soluble CD155 levels are significantly higher in patients with hepatocellular carcinoma than those in healthy donors, and these levels are positively correlated with tumor stage [12]. Serum soluble CD155 could be used as a predictive marker in the adjuvant diagnosis of diffuse large B-cell lymphoma [13]. Whether CD155 has significant changing in PE patients deserves further investigation.

Many recent reports, especially in the field of tumor immunology, have investigated the expression of the immune checkpoint molecules TIGIT, CD226 and CD155 and their possible role in immune regulation, while fewer papers have been published in the context of pregnancy. We have also found an imbalance of TIGIT and CD226 in recurrent spontaneous abortions in the early stages of the disease [14]. As a syndrome of pregnant disease, the involvement of these three molecules in the development of PE during pregnancy remains poorly understood. Therefore, we hope that the present study will provide data to support the understanding of TIGIT, CD226 and CD155 in PE patients.

## Objects and methods

### Study subjects

Written informed consent was obtained from all cases and controls with the approval of the Ethics Committee of the Obstetrics and Gynecology Hospital of Fudan University (no. 2021 − 214). Cases and controls were obtained from the Obstetrics and Gynecology Department of the Obstetrics and Gynecology Hospital of Fudan University from January 2022 to January 2024.

Patients were screened according to the American College of Obstetrics and Gynecology (ACOG) guidelines [14]. Among the rules for screening patients with PE: in addition to the features of new-onset hypertension and proteinuria after 20 weeks’ gestation, PE was considered severe if blood pressure was ≥ 160 mm Hg systolic or ≥ 110 mm Hg diastolic; or if at least one of the following clinical signs was present: renal insufficiency, pulmonary oedema, microvascular disease, thrombocytopenia, hepatic impairment, and severe peripheral organ involvement (visual disturbances and headache). Patients were considered to have mild PE if they met the diagnostic criteria for PE but not for severe PE. Patients with other obstetric or systemic conditions were excluded from the study, including pregnancies with chronic hypertension, pre-existing kidney disease, immune disorders, and HELLP (hemolysis, elevated liver enzymes, low platelet count) syndrome.

Pregnant women with normal pregnancy tests at the same time were recruited. Enrolled controls had normal blood pressure, no immune system diseases. Records were collected and matched, including age, medical history, gestational age, parity and maternal pre-pregnancy weight. Other known medical complications such as reproductive tract infections, immune disorders and genetic disorders that could affect the biomarker levels tested were also excluded from the PE patients and controls.

## Methods

We used blood samples taken from participants during normal medical examinations, without adding additional burden to the patient’s blood drawing. Venous blood from PE patients and controls was anticoagulated with EDTA-K_2_. The expression of TIGIT, CD226 and CD155 on the surface of different lymphocytes was detected by flow cytometry, and the level of CD155 in peripheral blood serum was measured by ELISA.

### Instruments and reagents

The flow cytometer was CytoFLEX (Beckman-Coulter, USA) and the analysis software was CytExpert, which was supplied with the instrument. Reagents: Antibodies were purchased from BD BioLegend (San Diego, USA), including CD3 PE-cy7, CD4 FITC, CD25 PE, CD8 BV650, CD127 APC, CD155 PE, TIGIT BV605, and CD226-BV785. Enzyme-linked immunosorbent assay ELISA (ELK, China) was performed according to the instructions and all samples were duplicated.

### Statistical methods

GraphPad Prism version 5.0 (GraphPad, USA) was used for the main statistics and graphs in this study. In this study, SPSS 19 was mainly used for normality analysis. Normally distributed variables were expressed as mean ± standard deviation (X ± SD), and non-normally distributed data were expressed as median and extreme values. Variables that were normally distributed between the two data sets were analyzed using the t-test, the Mann-Whitney U-test for skewed distribution parameters, and the chi-squared test for categorical variables.

## Results

### Clinical and demographic characteristics

A total of 24 cases in the PE group and 24 cases in the control group were included in this study. Relevant clinical data such as age and gestational week are shown in Table 1.

**Table 1:** Clinical data of the subjects in the PE and control groups

		Control group		PE group	
Number		24		24	
Age (years)		32.04 ± 4.03		31.67 ± 4.65	
Gestational age (weeks)		36.17 ± 3.94		35.58 ± 3.63	
Parity		0(0–2)		0(0–1)	
Gravidity		2(1–3)		1(1–3)	

### Difference in Treg expression of peripheral blood cells between the two groups

**Fig. 1:**
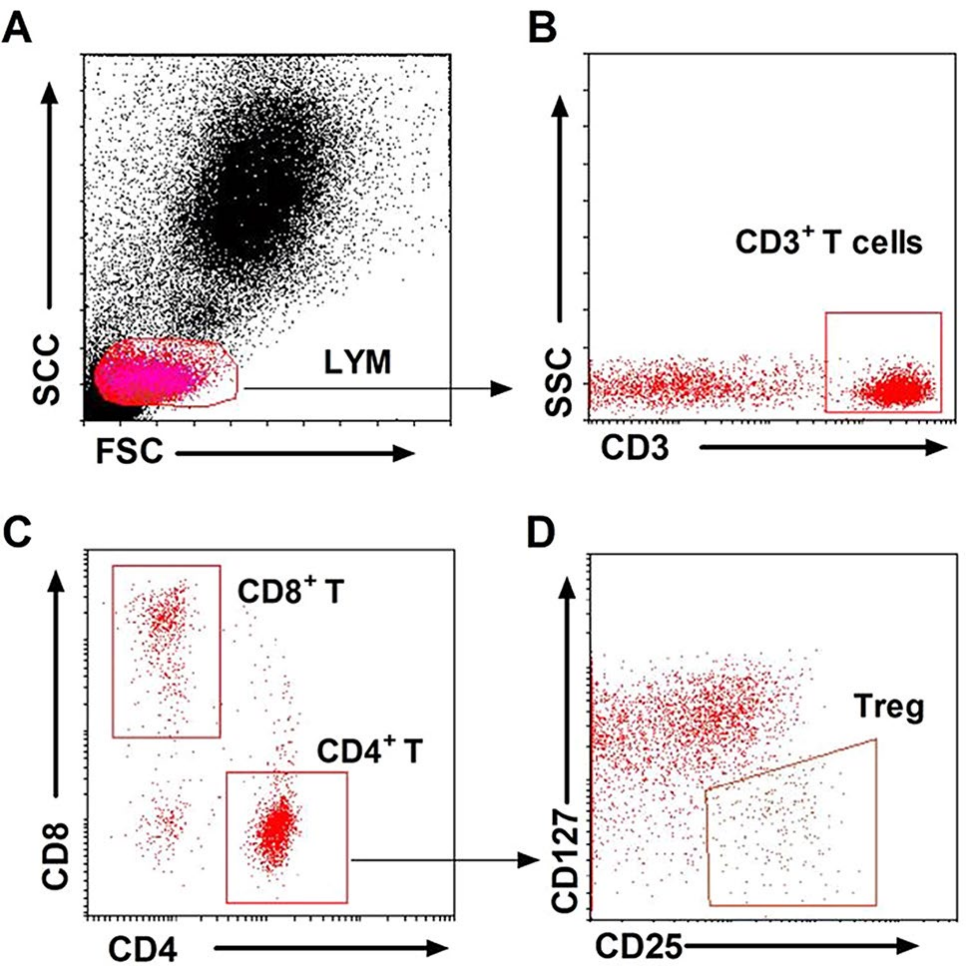
Different gating strategies for lymphocyte flow cytometry. *Note* Lymphocyte cells were gated according to forward scatter (FSC) and side scatter (SSC) **(A)**, CD3^+^ T cells were gated from lymphocyte cells **(B)**, CD4^+^ and CD8^+^ T cells were gated from CD3^+^ T cells **(C)**, and Treg were gated from CD4^+^ T cells **(D)**

The results of the proportions of CD4^+^, CD8^+^ T cells and CD25^+^ CD127^low^ Treg cells in the peripheral blood in the two groups are shown in Table 2, and the flow gating strategy is shown in Fig. 1. Among CD4^+^ and CD8^+^ T cells in the two groups, the difference was not statistically significant (*P* > 0.05); CD25^+^ CD127^low^ Treg cells in CD4 + T cells in the PE group was 6.43 ± 1.77%, which was significantly lower than that of the control group, which was 7.48 ± 1.71%, and the difference was statistically significant (*P* = 0.0420).

**Table 2:** Comparison of different lymphocytes in PE and control groups

			Control group		PE group	P value
CD4^+^ /CD3^+^T cell (%)			47.52 ± 10.58		52.49 ± 6.91	0.0604
CD8^+^ /CD3^+^T cell (%)			34.87 ± 8.41		33.30 ± 7.74	0.5044
CD25^+^CD127^low^/CD4^+^ T cell (%)			7.48 ± 1.71		6.43 ± 1.77	0.0420

*Note* Parameters in the table are expressed as mean ± standard deviation and data between two groups were analyzed by t-test. The expression of three molecules on peripheral blood immune cells was tested by flow cytometry and the level of CD155 in serum was tested by ELISA

### Expression of TIGIT on peripheral blood cells in the two groups

**Fig. 2:**
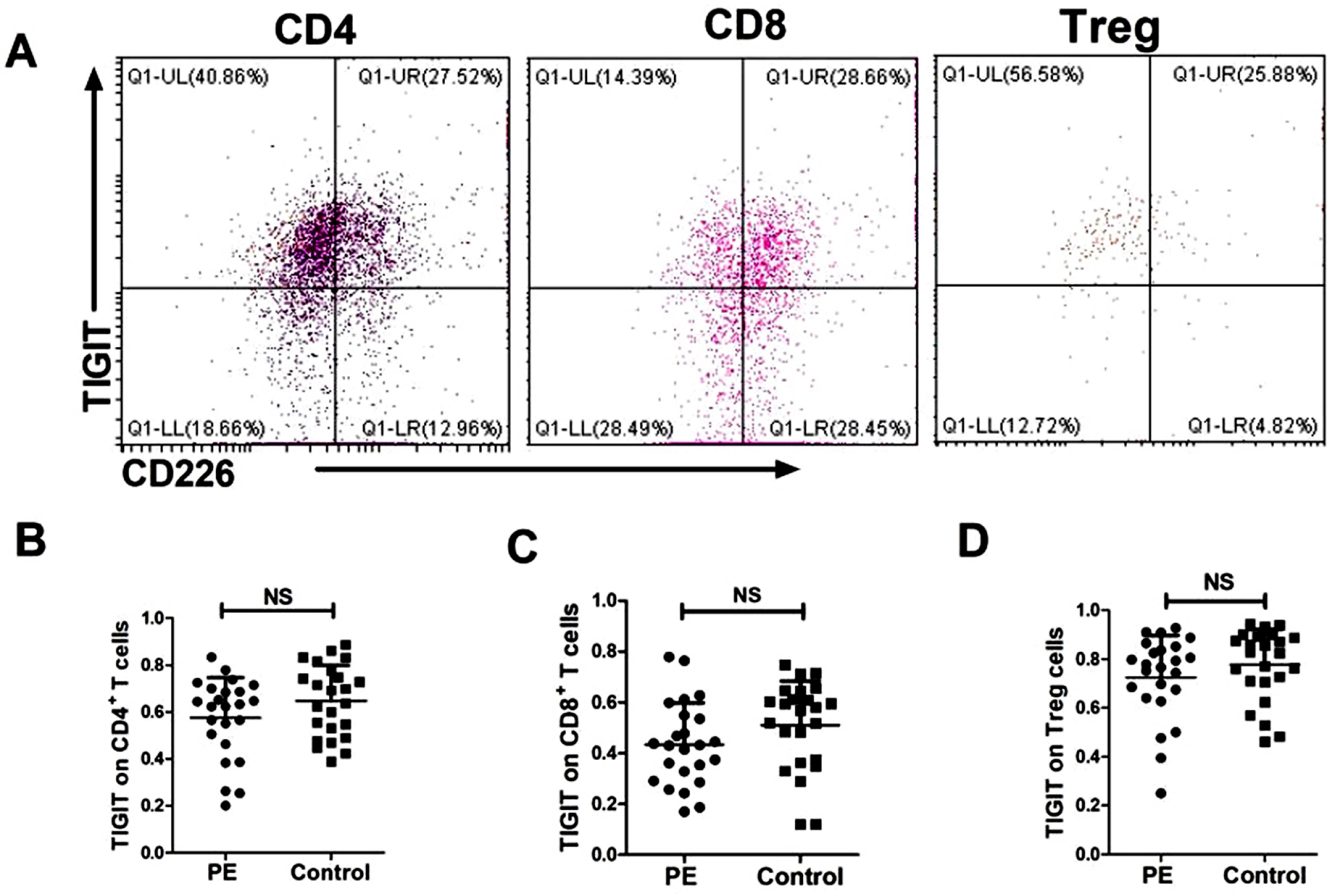
Expression of TIGIT and CD226 on peripheral blood cells in the two study groups. *Note* TIGIT and CD226 were gated from different T cells **(A)**. The TIGIT on CD4^+^**(B)**, CD8^+^ T cell **(C)** and Treg cells **(D)** surface were gated

The proportion of TIGIT on CD4^+^ T cells in the peripheral blood of PE patients was 57.58 ± 17.09%, and 64.73 ± 15.19% in the two group, with no statistically significant difference (*P* = 0.1322); the proportion of TIGIT on CD8^+^ T cells in the two groups was 43.43 ± 16. 39% and 51.01.36 ± 17.45%, respectively, with no statistically significant difference (*P* = 0.1280); the proportion of TIGIT on Treg cells in the two groups was 72.47 ± 17.23% and 77.60 ± 14.68% (*P* = 0.2725), and the results are shown in Fig. 2.

### Expression of CD226 on peripheral blood cells in the two groups

**Fig. 3:**
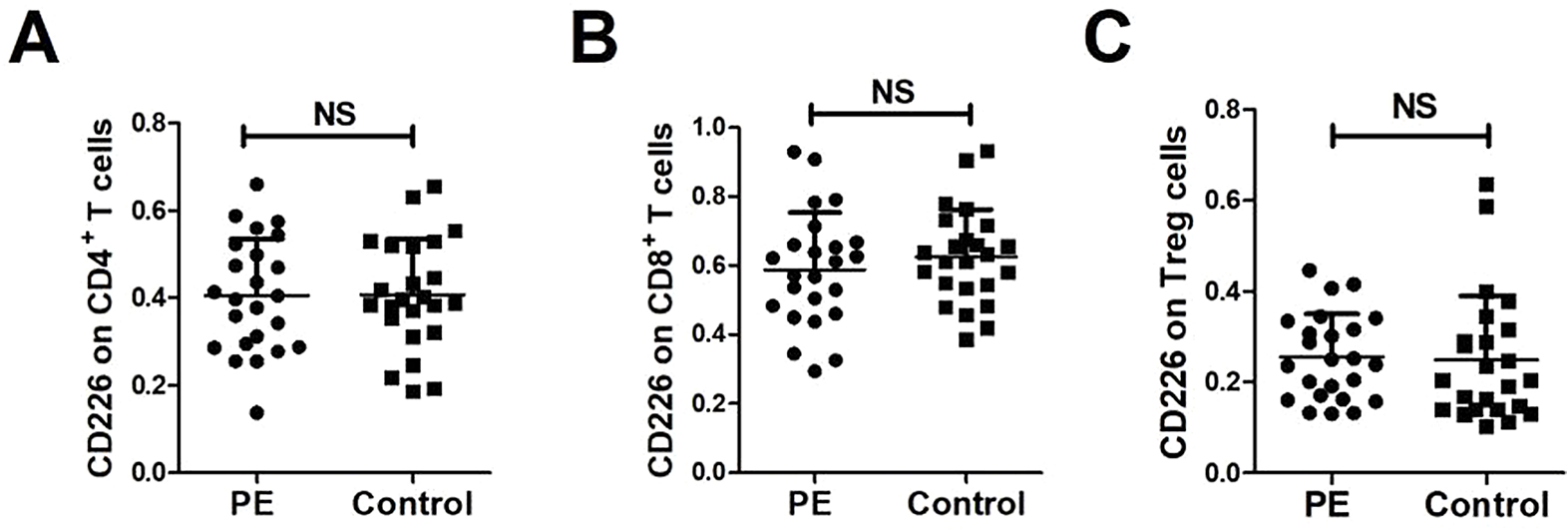
Expression of CD226 on peripheral blood cells of subjects in both groups. *Note* The CD226 on CD4^+^**(A)**, CD8^+^ T cell **(B)** and Treg cells **(C)** surface were gated

The proportion of CD226 on CD4^+^ T cells in the two groups was was 40.54 ± 13.04% and 40.74 ± 12.66%, with no statistically significant difference (*P* = 0.9579); the proportion of CD226 on CD8^+^ T cells in the two groups was 58.77 ± 16. 58% and 62.45 ± 13.74%, respectively, with no statistically significant difference (*P* = 0.4063); the proportion of CD226 on Treg cells was 25.47 ± 9.47% and 24.89 ± 14.10% in the two groups, with no statistically significant difference (*P* = 0.8672), and the results are shown in Figure 3.

### Expression of CD155 in serum between the two groups

As our previous results, the proportion of CD155 on CD4^+^ T cells in the peripheral blood of PE patients was 1.14 ± 0.90%, which was significantly higher than that of 0.94 ± 0.39% in the control group, with a statistically significant difference (*P* < 0.05); and the proportion of CD155 on CD8^+^ T cells in the two groups was 0.95 ± 0.36% and 0.79 ± 0.40% by flow cytometry, *P* > 0.05. However, such a low expression may no longer be suitable for detection and comparison by flow cytometry. Therefore, we determined the serum levels of CD155 in the two groups by ELISA. The level of CD155 in peripheral serum of PE patients was 6.64 ± 1.79 ng/ml, which was not significantly different from that in the control group 5.61 ± 1.77 ng/ml, *P* = 0.0505.

## Discussion

The involvement of immune checkpoint molecules, consisting of TIGIT/CD226 and CD155, in the immune regulation of the organism in physiological and pathological states has been increasingly recognized. In the physiological state, CD155 is more highly expressed on antigen-presenting cells and is involved in various processes such as cell adhesion, migration and proliferation [15]; in the pathological state, it can be overexpressed on tumor cells, promoting tumor proliferation, invasion and immune escape; meanwhile, it can act as a ligand and bind to the receptor molecules, TIGIT and CD226, with the function of regulating the immunity of T cells and NK cells [16]. PE is a pregnancy-related disorder characterized by hypertension and proteinuria, often accompanied by organ dysfunction, neurological symptoms or uteroplacental dysfunction [17]. As the main therapy for PE is medically induced premature labour, it is crucial to investigate its etiology, monitor high-risk pregnancies and apply early prediction models for the prevention and future management of this condition. During pregnancy, the immune system at the maternal-fetal interface develops a unique immune state that tolerates healthy fetal development, whereas its immune abnormalities can have serious consequences. In patients with PE, the immune balance between mother and fetus is altered, which can affect both intrinsic and adaptive immunity [18]. The expression, role and value of the immune checkpoint pathway in PE patients, is still little to be known.

In the present study, firstly we compared the expression differences between several common lymphocytes in the two groups, then compared the surface expression of the immune checkpoint molecules TIGIT, CD226 and CD155 on CD4^+^ and CD8^+^ T cells as well as on a subset of Treg cells, and finally examined the serum levels of CD155. We found no significant difference in the expression of CD4^+^ and CD8^+^ T cells between the two groups. However, the proportion of Treg cells was significantly lower in the PE group than that in the control group, which is similar to some reports in both PE patients and mice [19, 20]. The role of Treg in maternal-fetal immunity is critical for fetal protection against organ rejection, whereas a decrease in their number or abnormal function can cause adverse pregnancy outcomes [14]. Our results show that there is a decrease of Treg in the number in PE patients.

CD4^+^ T lymphocytes are important helper cells at the maternal-fetal interface and are composed mainly of subpopulations of TH1, TH2, TH17, and Treg cells, with the TH2 and Treg populations generally believed to play a more immune-tolerant role. Immune checkpoint molecules are involved in both the differentiation and function of CD4^+^ T cells, such as TIGIT and CD226 on the surface of most T lymphocytes and NK cells, particularly on activated lymphocytes [21]. The fact that CD155 has a higher affinity for TIGIT than CD226 highlights the predominance of inhibitory over activated signals [22]. Our results show that both TIGIT and CD226 are not expressed differently on CD4^+^ T cells in patients with PE and in controls. We speculate that the TIGIT, which plays roles in the inhibitory capacity of CD4^+^ T cells and participating in maternal tolerance of the fetus, maybe not change in the late phase of PE. It has been reported that after CD155-TIGIT binding, human dendritic cells secrete increased IL-10 and decreased IL-12, which may contribute to further down-regulation of T-cell activation [23]. One study found a significant reduction in TIGIT inhibitory expression in CD8^+^ T cells and a decrease in CD226 expression in PE patients [18], whereas our experimental results showed that these two molecules were not significantly altered on CD4^+^, CD8^+^ T cells and CD25^+^ CD127^low^ Treg cells. We speculate that the immune checkpoint molecule might contribute to the compensation or development during early-onset PE, or might play a greater role locally at the maternal-fetal interface but not show a difference in peripheral blood. We assume that the population studied by their team were patients with early onset PE at around 30 weeks’ gestation, whereas our current study focused mainly on patients at around 36 weeks’ gestation, during which time compensation or recovery did not result in any significant abnormality in the expression of CD226 on the surface of T cells in the periphery. The reduction of Treg, not these two molecules on Treg cells, suggests maybe just a reduction in the number of Treg cells but not a possible functional abnormality in patients with PE.

​ As a ligand, CD155 expression on the surface of T lymphocytes is generally low. As serum soluble CD155 is significantly elevated in some tumors and positively correlates with disease progression [15], it can be considered as a potential biomarker for the complementary diagnosis of tumor progression. Our results indicate no significant increase in peripheral serum in patients with PE, suggesting that peripheral serum CD155 may also be not correlated of PE. And there is no significant difference in the expression of CD155 in the serum of patients with mild and severe PE, which is somewhat different from our initial hypothesis; we speculate that the severity of PE is not positively proportional to the level of CD155 in serum, or that this statistical analysis did not adequately reflect the level of difference due to the limited sample size.

Our previous study confirmed the potential role of TIGIT and CD226 in maternal-fetal tolerance in recurrent spontaneous abortion [14], showing that peripheral blood Treg of patients with recurrent spontaneous abortion showed a decrease in TIGIT and an increase in CD226, which is still somewhat different from the results in PE. This cross-sectional comparison suggests that different changes in TIGIT/CD226 checkpoint molecules during pregnancy may be associated with different pregnancy complications. The mechanism of recurrent spontaneous abortion is different from PE, while these two diseases also have something in common, for their occurrence is related to the abnormality of maternal fetal interface. From the perspective of the expressions of TIGIT and CD226, it may provide new ideas for the occurrence of these two pregnancy-related diseases. The drawback of this study is the relatively small sample size, and we have not analyzed the expression and roles of these molecules in maternal-fetal interface. We are also in the process of collecting peripheral blood from PE patients and isolating mononuclear cells to provide a reserve for follow-up mechanistic studies and clinical modeling applications. However, we found the CD155, TIGIT and CD226 expression in fresh blood were different from that in PBMC stored in the freezer at minus 80 degrees Celsius before the experiment. The TIGIT and CD226 showed lower expressiosn on T cells cryopreserved in minus 80 degrees Celsius than that on freshly collected blood. For early detection of preeclampsia and early intervention, it is more meaningful for clinical treatment. Unfortunately, the specimen we tested was from a patient at the time of preeclampsia. However, we did not find a suitable time to collect the specimen, which is also worth thinking about. This study might provide the preliminary data basis for the further mechanism research.

## Conclusions

Immune checkpoint molecules maybe involved in maternal-fetal immunity during pregnancy. However, the present results demonstrate that no alteration of TIGIT, CD226 and CD155 are present in the peripheral blood of patients with PE.

## Data Availability

The data in the current study are available from the corresponding author after published on reasonable request.

## Abbreviations

PE: preeclampsia
TIGIT: T cell immunoglobulin and immunoreceptor tyrosine-based inhibitory motif domains
ACOG: American College of Obstetrics and Gynecology
Treg: regulatory T cell
HELLP: hemolysis, elevated liver enzymes, low platelet count
TH: helper T cell
